# Patterns of medication use among individuals with Covid-19 in Brazil: Epicovid 2.0

**DOI:** 10.11606/s1518-8787.2026060007542

**Published:** 2026-07-31

**Authors:** Romina Buffarini, Cesar Gomes Victora, Pedro Curi Hallal, Mariângela Freitas da Silveira

**Affiliations:** I Universidade Federal de Pelotas. Programa de Pós-graduação em Epidemiologia. Pelotas, RS, Brasil; II Universidade Federal de Pelotas. Centro Internacional de Equidade em Saúde. Pelotas, RS, Brasil; III University of Illinois Urbana-Champaign. Department of Health and Kinesiology. Urbana, IL, United States

**Keywords:** SARS-CoV-2, Coronavirus, Epidemiology, Drug Use, Cross-Sectional Study

## Abstract

**OBJECTIVE:**

To describe the patterns of medication use among individuals with Covid-19 to treat the disease in Brazil, according to area of residence, demographic and socioeconomic characteristics, religion, and level of trust in scientists.

**METHODS:**

Epicovid 2.0 National Survey (2024) in 133 municipalities. The analysis included participants who reported having had an episode of Covid-19. The use of antibiotics, azithromycin, antipyretics, chloroquine, and ivermectin was assessed, in addition to the composite outcomes: use of chloroquine and/or ivermectin; chloroquine, ivermectin, and/or azithromycin (CIA); and use of any medication. The frequency of use and 95% confidence intervals were estimated, considering the complex sampling design and sample weighting

**RESULTS:**

The frequency of use of any medication to treat Covid-19 was 63.0% (95%CI 60.0–65.9). Antipyretics were the most commonly used (53.7%), followed by antibiotics (36.2%) and azithromycin (27.2%). The isolated use of ivermectin was reported by 23.6% and that of chloroquine by 12.1%; the use of ivermectin and/or chloroquine reached 27.8% and that of CIA, 39.0%. Overall, medication use was more frequent in the North, Northeast, and Central-West regions and among women. Use was concentrated among adults aged 40–59, people with low educational attainment, and those with lower incomes. Self-identified black and brown individuals reported higher use of chloroquine. Use of chloroquine, ivermectin, and combinations of these medications was more common among evangelicals (0.05 < p < 0.1) and among people who consulted doctors (p < 0.001). Participants who reported distrusting scientists reported higher use of chloroquine and chloroquine/ivermectin compared to those who reported trusting scientists.

**CONCLUSION:**

Four out of ten people who reported episodes of Covid-19 consumed medications not recommended by science to treat the disease. Medication use tended to be more frequent among women, in the northern half of Brazil, among evangelicals, and among people with lower income and education levels. These groups should be prioritized in future campaigns on the appropriate use of medications.

## INTRODUCTION

Medications are central components of healthcare practices, used in the prevention and treatment of diseases. However, their use may be associated with significant adverse effects when administered inappropriately^1^. At the onset of the Covid-19 pandemic, in the absence of effective therapies for a previously unknown virus, various medications with some biological plausibility for treating the disease’s complications began to be tested and used in clinical practice through therapeutic repositioning strategies^2,3^. In this context, off-label use was adopted in different clinical settings. Advances in the evidence, however, began to demonstrate the lack of benefit for most of these interventions^4^.

Despite this, in the months following the onset of the pandemic, there was widespread use of both prescription and over-the-counter medications^8,9^, often without scientific basis^10^, raising concerns about the irrational use of medications and potential individual and collective risks^11^. In addition to medication use practices, such as frequent self-medication, already described in the country prior to the pandemic^12,13^, a scenario emerged marked by the widespread dissemination of non-evidence-based information regarding supposed treatments for the disease^14^.

Analyses of disinformation patterns on social media identified Brazil as one of the countries with the highest prevalence of false or misleading content regarding recommended medications throughout the pandemic^14^, suggesting low incorporation of scientific evidence into the public debate. While national and international scientific institutions rejected the use of various medications (such as chloroquine, ivermectin, and azithromycin)^5,7,15^, their use was encouraged by the President of the Republic and the Ministry of Health^20,21^, and supported by medical entities, evangelical religious leaders, and numerous digital influencers^22^, framing misinformation as a strategy linked to political disputes in the fight against the epidemic^25^. The academic literature shows that the promotion of these drugs was not accidental but constituted a political strategy for managing the pandemic. By offering a “cure,” the promoting groups—whether governmental, medical, media, or religious—sought to avoid social isolation measures and shift individual responsibility for care, creating a false sense of security among the population.

Despite the relevance of this context, representative national data on patterns of consumption of these medications during the pandemic remain scarce. The existing evidence is based, for the most part, on specific populations (e.g., students, nurses, teachers)^29^, or studies with participants from the general population featuring small samples and heterogeneous sampling designs^32,33^, limiting the generalizability of the findings at the population level.

Given this gap, the objective of this study was to describe the patterns of use of different medications (recommended and off-label) that individuals who reported having had Covid-19 in Brazil used to treat the disease, according to region of residence, demographic and socioeconomic characteristics, religious affiliation, and trust in scientists, using data from the population-based national survey Epicovid 2.0.

## METHODS

This study used data from the second edition of the Epicovid National Survey (Epicovid 2.0), conducted between March and June 2024 in all 27 federal units of Brazil. The sampling was identical to that of the original Epicovid study, conducted in four stages during 2020 to document the progression of the Covid-19 pandemic in the country. Methodological details can be found in another publication^34^.

The Instituto Brasileiro de Geografia e Estatística (IBGE - Brazilian Institute of Geography and Statistics) divides the national territory into 133 intermediate regions. In each of these, the most populous city was selected as the sentinel area. The eligible population consisted of residents of permanent private households located in the randomly selected urban census tracts. A multistage probability sampling process was used. First, 133 municipalities were selected; second, 25 urban sectors were selected with probability proportional to size; next, ten households were randomly selected within each sector, using official IBGE maps and lists. Finally, all residents present in the household were recorded, and one resident was randomly selected via a mobile app. In case of refusal, a second resident was selected at random, and if both refused, the team proceeded to the neighboring household located to the right of the original household, enumerating its residents and selecting one for the interview. In single-person households where there was a refusal, the next household was visited. The sampling plan resulted in a sample size of 33,250 individuals.

The interviews were conducted in person using a mobile app administered by a trained team hired by a company specializing in population surveys. Data collection took place from May to June 2024, simultaneously across all cities, thereby reducing temporal and seasonal biases.

### Variables

#### Outcomes

The outcomes were reports of medication use to treat the disease during the Covid-19 episode; information was obtained through specific questions about the use of each medication. The sample analyzed was restricted to individuals who reported having experienced one or more episodes of Covid-19. For each drug evaluated, the response was coded dichotomously (yes/no).

Five groups of medications were analyzed: any antibiotic, azithromycin, antipyretics (collected in the questionnaire as “medications for fever,” a term used to facilitate understanding among respondents), chloroquine, and ivermectin. Three composite outcomes were also analyzed: “use of chloroquine and/or ivermectin”, “use of chloroquine, ivermectin, and/or azithromycin”, referred to as CIA, and “use of any medication”. Therefore, the analyses focused on off-label medications, which gained widespread public attention during the pandemic despite the lack of scientific evidence of their efficacy in treating Covid-19, as per systematic reviews and clinical guidelines^2,4,6,35^. The composite outcomes for off-label medications were defined to capture both the isolated and combined use of these drugs, reflecting widely adopted practices in the context of the pandemic.

The composite outcome “use of any medication” was defined as the use of at least one of the following: antibiotics, antipyretics, anti-inflammatories/corticosteroids, chloroquine, ivermectin, or nirmatrelvir/ritonavir, and was used to represent the overall picture of medication use among individuals with Covid-19. The use of nirmatrelvir/ritonavir was initially investigated; however, due to the low frequency of its use (2.2%), this drug was not included in the analyses as an individual outcome, remaining only within the composite outcome “use of any medication”. The antibiotic azithromycin was investigated separately and was also included in the outcomes of ICU admission, use of any antibiotic, and use of any medication. Its separate analysis is justified because it was the most widely used antibiotic during the pandemic and is part of the group of medications widely used without scientific evidence of efficacy for treating Covid-19.

#### Exposures

Explanatory variables included geographic region of residence, sociodemographic characteristics, religious affiliation, trust in scientists, and seeking medical care. The region of residence was classified according to Brazilian macro-regions: North, Northeast, Central-West, Southeast, and South.

Sociodemographic variables included self-reported race/ethnicity, according to IBGE categories (white, black, brown, Asian, or indigenous); sex (female or male); age, categorized into age groups (0–19, 20–39, 40–59, 60–69, and 70 or older); and educational attainment in completed years of schooling (0—no formal education, 1–4, 5–8, 9–12, and 13 years or more). Socioeconomic status was estimated based on the household’s monthly income, expressed in minimum wages (less than 1, 1 to 3, 3 to 5, and 5 or more).

Religious affiliation was grouped into three categories: no religion, Catholic, and Evangelical. Other religions (Spiritism, Umbanda/Candomblé, Judaism, Islam, Buddhism, and others) were not analyzed separately due to the low individual frequency of each group (< 1%) and the lack of a conceptual basis for their grouping, which is why they were excluded from the specific analyses of this variable.

The variable “trust in scientists” was assessed using the following question: *“How much do you trust scientists regarding information about Covid-19?”* Responses were initially collected on a five-point ordinal scale: *never trust; rarely trust; neither trust nor distrust; moderately trust; always trust*. They were subsequently grouped into three categories: “Distrust” (never/rarely trust), “Neither trust nor distrust,” and “Trust” (moderately/always trust). Seeking medical care during the Covid-19 episode (yes/no) was also included.

#### Analyses

Descriptive analyses (absolute and relative frequencies) were performed. The frequencies and 95% confidence intervals (95%CI) of the eight outcomes were stratified according to the explanatory variables.

To verify the overlap in the use of off-label medications (chloroquine, ivermectin, and azithromycin), a Venn diagram was created, showing their combinations of isolated and concomitant use.

All analyses accounted for the complex sampling design (sample weights, sample stratified by municipality, and use of clusters within each municipality) using the *svy* command in Stata software. A detailed description of the calculation of weighted sample weights is presented in a specific publication of the Epicovid 2.0 protocol^34^, and all results presented below were weighted. There was missing data only for educational level (6%) and household income (7%). Participants with missing values for these variables were not included in the corresponding stratified analyses. All statistical analyses were performed in Stata, version 19.0 (StataCorp, College Station, United States).

#### Ethical Considerations

The study was approved by the Ethics Committee of the Escola de Educação Física da Universidade Federal de Pelotas (CAAE: 68382923.8.0000.5313). All adult participants signed an informed consent form. For minors, consent was obtained from the legal guardian.

## RESULTS

**Figure 1:**
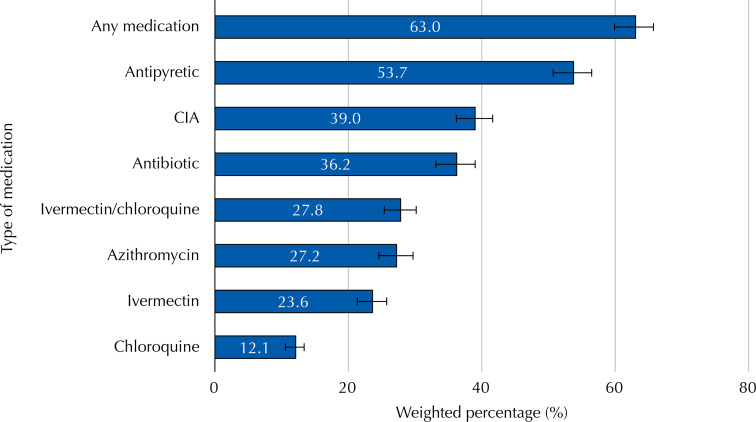
Weighted prevalences of medication use among participants reporting Covid-19. Epicovid 2.0, Brazil, 2024 (n = 9,591).

The analytical sample was restricted to 9,591 participants with a prior history of Covid-19, corresponding to 28.6% (95%CI 27.3–30.0) of the total sample^34,36^. Of these individuals, the majority reported only one episode (76.7%), while 17% reported two, 5% reported three, and 1% reported having experienced four or more episodes of Covid-19. The frequency of medication use was 63.0% (95%CI 60.0–65.9). The most commonly used medications were antipyretics (53.7%; 95%CI 50.8–56.6), followed by antibiotics (36.2%; 95%CI 33.3–39.2) and azithromycin (27.2%; 95%CI 24.7–29.9). The use of ivermectin alone was reported by 23.6% (95%CI 21.5–25.9) of participants, and that of chloroquine by 12.1% (95%CI 10.7–13.5). The use of ivermectin and/or chloroquine reached 27.8% (95%CI 25.5–30.3), while the composite outcome CIA (chloroquine, ivermectin, and/or azithromycin) had a frequency of 39.0% (95%CI 36.3–41.8) (Figure 1).

CIA: chloroquine, ivermectin, and/or azithromycin.

**Figure 2:**
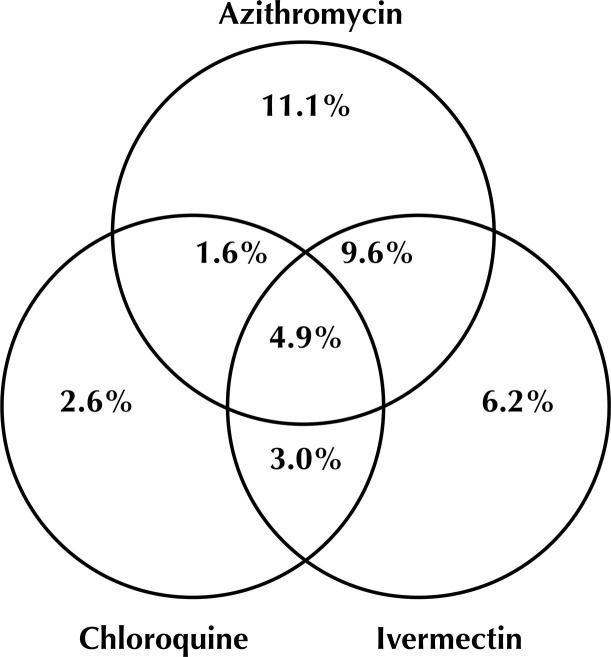
Overlap in the use of off-label medications (chloroquine, ivermectin, and azithromycin) among participants with Covid-19. Epicovid 2.0, Brazil, 2024 (n = 9,591).

The Venn diagram (Figure 2) shows the overlap in the use of chloroquine, ivermectin, and azithromycin, indicating that of the 40% of participants who used one of the off-label medications, 4.9% used all three.

The sample consisted mainly of residents of the Southeast (43.6%), followed by the Northeast (17.3%) and South (16.6%). Women accounted for 52.8% of the sample, and most participants were aged 20–39 years (38.4%) and 40–59 years (32.0%). Nearly half self-identified as white (47.1%), followed by 41.4% black and 10.6% brown, while the Asian and indigenous categories accounted for less than 1% of the sample. Regarding education, nearly 80% of participants had 9 or more years of formal education, and 2% reported having no formal education. Two-thirds of the sample reported earning up to 3 minimum wages, one-fifth reported 3 to 5 minimum wages (19.1%), and 13.9% earned 5 or more. Most participants identified as Catholic (39.4%) or Evangelical (35.0%); approximately 70% reported trusting scientists and having sought some form of medical care during their Covid-19 episode (Tables 1 and 2).

Overall, the use of all medications was more frequent in the North, followed by the Northeast and Central-West, with lower frequencies in the Southeast and South (p < 0. 0.001), except for the use of chloroquine, ivermectin, and the combined outcome of both of these drugs, for which higher frequencies were observed in the North and Central-West (Table 1). Women reported higher use of antibiotics, antipyretics, azithromycin, CIA, and any medication compared to men. The isolated use of chloroquine was more frequent among men, with a borderline difference (p = 0.056). Regarding age, the highest frequencies were concentrated in the 40–59 age group, with reduced use among younger and older individuals, although without a uniform pattern for all medications.

**Table 1:** Prevalence of medication use to treat Covid-19 during the pandemic by region, sex, and age (weighted results). Epicovid 2.0, Brazil, 2024 (n = 9,591).

Explanatory variables	n^a^ (%)	Antibiotics	Antipyretics	Chloroquine	Ivermectin	Azithromycin	Chloroquine/ivermectin	CIA	Any medication
		% 95%CI	% 95%CI	% 95%CI	% 95%CI	% 95%CI	% 95%CI	% 95%CI	% 95%CI
Region		p < 0.001	p = 0.008	p < 0.001	p < 0.001	p < 0.001	p < 0.001	p < 0.001	p < 0.001
North	1,770 (11.9)	52.3 (46.9–57.7)	63.4 (58.8–67.7)	19.9 (16.8–23.4)	32.7 (28.9–36.7)	41.9 (37.1–46.9)	37.6 (33.5–41.8)	54.0 (49.4–58.6)	74.1 (69.4–78.3)
Northeast	2,480 (17.3)	38.7 (35.5–42.0)	57.9 (54.0–61.7)	12.1 (10.0–14.6)	29.8 (26.8–33.1)	28.6 (25.7–31.6)	33.5 (30.4–36.7)	43.5 (40.1–47.0)	67.6 (63.9–71.1)
Central-West	1,042 (10.6)	36.6 (31.8–41.7)	53.1 (45.7–60.4)	17.6 (12.3–24.6)	31.8 (26.3–37.8)	26.0 (21.5–31.0)	37.2 (30.3–44.8)	43.6 (36.5–51.0)	65.2 (58.4–71.4)
Southeast	2,526 (43.6)	31.5 (25.6–38.2)	51.3 (45.3–57.2)	8.5 (6.5–11.2)	18.3 (14.2–23.3)	24.7 (19.5–30.7)	22.5 (18.1–27.6)	34.2 (28.8–40.1)	59.2 (53.1–65.1)
South	1,773 (16.6)	33.9 (30.7–37.3)	49.0 (45.8–52.2)	12.0 (10.2–14.2)	19.4 (17.0–22.2)	22.8 (20.1–25.7)	23.0 (20.5–25.8)	32.9 (30.0–35.9)	58.7 (55.0–62.4)
Sex		p = 0.003	p = 0.004	p = 0.056	p = 0.1	p < 0.001	p = 0.46	p = 0.002	p = 0.001
Male	3,293 (47.2)	31.9 (27.9–36.2)	49.4 (44.7–54.0)	13.5 (11.2–16.3)	21.5 (17.8–25.7)	21.8 (18.2–25.8)	26.8 (22.9–31.2)	34.4 (30.2–38.8)	58.0 (53.4–62.6)
Female	6,298 (52.8)	40.0 (36.3–43.9)	57.5 (54.2–60.8)	10.7 (9.4–12.3)	25.5 (23.1–28.0)	32.1 (29.0–35.5)	28.7 (26.1–31.5)	43.1 (39.7–46.5)	67.4 (63.9–70.7)
Age group (years)		p = 0.47	p = 0.004	p < 0.001	p = 0.003	p = 0.047	p = 0.001	p < 0.001	p = 0.125
0–19	658 (13.1)	35.1 (25.6–46.0)	56.3 (47.4–64.8)	7.1 (4.5–11.1)	20.9 (12.5–33.0)	26.5 (16.9–38.9)	24.6 (15.9–36.2)	37.3 (27.7–47.9)	62.8 (54.3–70.5)
20–39	2,844 (38.4)	34.9 (29.9–40.2)	54.8 (49.3–60.2)	9.5 (7.6–11.9)	18.9 (15.9–22.3)	24.8 (21.0–29.2)	23.0 (19.7–26.7)	34.2 (29.9–38.8)	62.0 (56.5–67.3)
40–59	3,557 (32.0)	39.6 (35.6–43.7)	57.2 (52.8–61.5)	16.7 (14.3–19.5)	31.7 (28.2–35.6)	32.9 (29.3–36.8)	36.3 (32.8–40.0)	47.7 (43.7–51.6)	67.3 (62.8–71.6)
60–69	1,551 (10.1)	33.1 (28.0–38.6)	45.4 (39.6–51.4)	13.6 (10.1–18.0)	21.9 (18.0–26.2)	25.1 (20.6–30.3)	27.0 (22.5–32.0)	37.1 (32.0–42.5)	58.0 (51.6–64.2)
≥ 70	978 (6.5)	34.1 (27.3–41.6)	37.3 (29.4–46.0)	11.6 (7.4–17.7)	19.6 (14.4–26.0)	18.2 (13.6–24.0)	22.1 (16.8–28.5)	30.6 (24.4–37.5)	55.4 (47.0–63.5)

95%CI: 95% confidence interval; CIA: chloroquine, ivermectin, and/or azithromycin.

^a^ Absolute frequency presented without weighting by sample weights.

Table 2 shows that race/skin color was associated only with the use of chloroquine (p = 0.001), with higher consumption among black and brown individuals compared to the other groups. Regarding education, higher frequencies of medication use were concentrated among individuals with 5 to 8 years of schooling, specifically for the use of antibiotics, chloroquine, chloroquine/ivermectin, and any medication (p < 0.05) (Table 2). Regarding household income, an inverse association was identified, with a decrease in use as income increased, particularly for antipyretics (p = 0.003), chloroquine (p = 0.032), and any medication (p = 0.005). A similar pattern was observed for the other medications, although without statistical significance.

**Table 2:** Prevalence of medication use to treat Covid-19 during the pandemic by socioeconomic characteristics, religion, trust in scientists, and seeking medical care (weighted results). Epicovid 2.0, Brazil, 2024 (n = 9,591).

Explanatory variables	n^a^ (%)	Antibiotics	Antipyretics	Chloroquine	Ivermectin	Azithromycin	Chloroquine/ivermectin	CIA	Any medication
		% 95%CI	% 95%CI	% 95%CI	% 95%CI	% 95%CI	% 95%CI	% 95%CI	% 95%CI
Race		p = 0.476	p = 0.6	p = 0.001	p = 0.674	p = 0.741	p = 0.334	p = 0.655	p = 0.159
White	3,340 (47.1)	34.9 (30.3–39.7)	54.7 (50.5–58.8)	9.5 (7.9–11.4)	22.9 (19.0–27.2)	26.2 (22.2–30.6)	26.3 (22.3–30.7)	37.7 (33.5–42.2)	64.8 (60.8–68.6)
Black	4,412 (41.4)	38.2 (34.5–42.0)	52.9 (48.7–57.1)	15.0 (12.7–17.5)	24.8 (22.3–27.6)	28.1 (25.2–31.1)	30.0 (26.9–33.3)	40.4 (36.9–44.0)	62.0 (57.7–66.2)
Brown	1,244 (10.6)	34.1 (27.9–40.9)	51.5 (44.6–58.3)	12.2 (8.4–17.3)	22.2 (17.9–27.1)	28.5 (22.4–35.4)	26.3 (21.2–32.1)	39.2 (32.5–46.3)	57.9 (51.0–64.4)
Yellow	204 (0.4)	34.9 (22.7–49.5)	50.8 (34.6–66.9)	9.2 (4.8–17.0)	21.7 (12.9–34.2)	23.5 (14.6–35.6)	24.4 (15.0–37.1)	31.0 (20.1–44.4)	68.5 (52.1–81.3)
Indigenous	132 (0.5)	40.8 (23.0–61.3)	68.2 (49.0–82.7)	14.7 (6.6–29.8)	25.0 (12.9–43.0)	32.0 (17.6–51.0)	27.4 (14.5–45.6)	36.9 (20.7–56.8)	77.8 (60.2–89.1)
Years of schooling		p = 0.018	p = 0.081	p = 0.004	p = 0.06	p = 0.392	p = 0.005	p = 0.07	p = 0.008
No education	301 (2.0)	43.0 (32.0–54.7)	60.6 (49.2–70.9)	14.1 (8.9–21.4)	21.5 (14.7–30.2)	29.2 (20.6–39.7)	27.3 (19.4–36.9)	38.4 (28.5–49.4)	67.8 (56.7–77.2)
1 to 4	919 (6.2)	34.2 (28.3–40.6)	52.4 (44.9–59.7)	12.4 (8.5–17.7)	16.9 (13.1–21.6)	22.2 (17.2–28.0)	22.4 (17.4–28.3)	34.0 (27.8–40.8)	59.6 (51.9–66.8)
5 to 8	1,774 (13.7)	46.3 (38.6–54.2)	61.0 (54.3–67.3)	16.0 (12.1–20.8)	30.0 (21.8–39.7)	32.0 (23.9–41.4)	35.6 (27.4–44.7)	46.8 (38.9–54.8)	72.3 (66.7–77.3)
9 to 12	4,556 (50.9)	35.2 (31.9–38.6)	54.0 (50.3–57.6)	12.9 (10.9–15.2)	24.5 (21.8–27.4)	27.0 (24.1–30.1)	29.6 (26.6–32.8)	39.3 (36.0–42.8)	63.0 (59.1–66.7)
≥ 13	1,984 (27.2)	33.4 (27.5–39.8)	49.9 (43.6–56.2)	8.4 (6.5–10.8)	20.8 (17.0–25.2)	26.7 (21.8–32.3)	22.3 (18.4–26.7)	36.1 (30.8–41.7)	59.4 (53.7–64.9)
Household income (minimum wage)		p = 0.056	p = 0.003	p = 0.032	p = 0.842	p = 0.298	p = 0.174	p = 0.197	p = 0.005
< 1	2.672 (24.5)	42.0 (36.5–47.7)	61.9 (57.1–66.6)	13.7 (11.1–16.8)	24.0 (20.5–27.9)	31.0 (25.9–36.6)	28.7 (24.6–33.3)	43.1 (37.8–48.5)	70.1 (65.5–74.3)
1 to 3	3,891 (42.5)	36.6 (32.0–41.6)	54.4 (49.8–59.0)	13.5 (11.0–16.5)	23.9 (20.0–28.4)	27.4 (23.3–31.9)	30.0 (25.8–34.6)	39.8 (35.5–44.1)	63.8 (59.2–68.2)
3–5	1,510 (19.1)	33.5 (28.2–39.2)	50.2 (43.7–56.6)	10.7 (8.4–13.5)	24.2 (19.6–29.5)	24.5 (20.1–29.5)	26.8 (22.2–32.0)	36.0 (30.8–41.5)	58.6 (52.1–64.9)
≥ 5	927 (13.9)	30.0 (23.0–38.0)	44.8 (36.7–53.2)	8.1 (6.0–11.0)	21.3 (16.9–26.4)	24.1 (17.5–32.2)	22.4 (17.9–27.6)	34.7 (27.6–42.6)	55.1 (46.9–63.1)
Religion		p = 0.123	p = 0.454	p = 0.056	p = 0.058	p = 0.292	p = 0.08	p = 0.08	p = 0.627
None	1,646 (25.6)	31.3 (24.3–39.1)	53.9 (46.7–60.9)	10.0 (7.2–13.5)	18.8 (13.1–26.4)	23.3 (16.8–31.4)	24.4 (18.1–31.9)	33.3 (26.3–41.1)	62.3 (55.4–68.7)
Catholic	4,316 (39.4)	37.6 (33.8–41.5)	51.6 (47.8–55.4)	12.2 (10.3–14.5)	23.6 (20.8–26.7)	28.1 (24.7–31.8)	27.0 (24.0–30.2)	40.1 (36.4–43.9)	62.4 (58.5–66.2)
Evangelical	3,058 (35.0)	38.8 (34.9–42.9)	55.8 (51.2–60.3)	15.1 (12.3–18.3)	27.7 (24.6–31.0)	29.0 (25.5–32.7)	32.6 (28.8–36.6)	42.3 (38.0–46.8)	65.2 (60.7–69.4)
Trust in scientists		p = 0.136	p = 0.41	p < 0.001	p = 0.349	p = 0.751	p = 0.022	p = 0.232	p = 0.199
Trust	6,283 (70.7)	34.6 (30.8–38.6)	53.9 (50.1–57.7)	10.1 (8.7–11.8)	22.8 (19.9–25.9)	26.8 (23.3–30.6)	26.2 (23.2–29.4)	37.9 (34.2–41.6)	62.3 (58.5–66.0)
Neither trust nor distrust	1,434 (13.9)	39.9 (33.2–47.1)	50.0 (43.0–57.0)	12.4 (9.4–16.3)	25.5 (20.9–30.7)	28.4 (23.3–34.1)	29.3 (24.3–34.8)	39.6 (33.6–45.9)	60.8 (53.5–67.6)
Distrust	1,874 (15.4)	40.1 (35.7–44.7)	56.0 (51.1–60.7)	20.6 (16.8–25.0)	25.7 (22.6–29.2)	28.4 (24.9–32.2)	34.0 (29.5–38.9)	43.5 (39.0–48.2)	68.0 (63.2–72.5)
Seeking medical care		p < 0.001	p < 0.001	p < 0.001	p < 0.001	p < 0.001	p < 0.001	p < 0.001	p < 0.001
No	3,021 (30.9)	21.2 (18.2–24.7)	36.6 (32.4–41.1)	8.3 (6.5–10.6)	16.4 (14.0–19.2)	16.4 (13.6–19.7)	18.8 (16.1–21.8)	26.1 (22.6–29.9)	46.1 (41.6–50.7)
Yes	6,750 (69.1)	42.9 (39.2–46.6)	61.3 (58.1–64.4)	13.7 (12.0–15.6)	26.8 (24.0–29.9)	32.1 (28.9–35.4)	31.9 (28.9–35.0)	44.7 (41.6–47.9)	70.5 (67.4–73.5)

95% CI: 95% confidence interval; CIA: chloroquine, ivermectin, and/or azithromycin; SM: minimum wage.

^a^ Absolute frequency presented without weighting by sample weights.

Regarding religion, higher frequencies were observed among evangelical individuals for most of the medications analyzed, although associations approaching statistical significance were observed only for chloroquine (p = 0.056), ivermectin (p = 0.058), and for the combined outcomes (p = 0.08).

Individuals who reported distrusting scientists had a frequency of chloroquine use approximately twice as high (p < 0.001) and a frequency of chloroquine/ivermectin use 1.3 times higher (p = 0.022), compared to those who reported trusting scientists. The use of all medications was higher among individuals who sought medical care during their Covid-19 episode (p < 0.001).

## DISCUSSION

Due to its national scope, the present study provides the most comprehensive overview of medication use during Covid-19 episodes in Brazil, and possibly the most extensive study worldwide. Our objective was to measure patterns of use according to various stratifying variables, using simple and descriptive analyses, with the aim of informing health policies for future epidemics. For this reason, there was no indication for more complex analyses or multivariate statistical models.

Our results revealed regional, sociodemographic, and behavioral differences in the use of recommended medications—as well as off-label medications—among participants who reported having Covid-19. Nearly two-thirds used at least one type of medication, and about 40% used one or more off-label medications such as chloroquine, ivermectin, and/or azithromycin.

In general, the patterns of medication use by region, sex, and age group identified in this study are consistent with the Brazilian literature on self-medication for acute conditions^12,37^. The population-based study: Pesquisa Nacional sobre Acesso, Utilização e Promoção do Uso Racional de Medicamentos (PNAUM – National Survey on Access, Use and Promotion of Rational Use of Medicines), conducted between September 2013 and October 2014, revealed in various analyses higher medication consumption among women, as well as significant regional and socioeconomic inequalities in the use of these medications. The higher use observed in the Northeast, Central-West, and North regions mirrors the findings of the PNAUM and may reflect regional inequalities in access to health services^12,37^, with greater self-medication in contexts of lower availability of formal care. Similarly, the higher consumption of medications among lower-income individuals observed in our study may be influenced by reduced access to timely medical care among socioeconomically more vulnerable groups.

Women reported higher use of most of the medications analyzed, a finding already described in previous PNAUM analyses^12,37^; the only exception was the higher use of chloroquine among men, which may be related to aspects of the promotion of this drug, which will be discussed below. Regarding age, consumption of all categories of medications analyzed was highest among adults aged 40 to 49; this finding is consistent with the PNAUM analysis that assessed self-medication in Brazil and showed that the prevalence of self-medication was higher in the 20–39 and 40–59 age groups compared to the extreme age groups^12^. This finding may reflect differences in the use of health services, since older individuals generally receive more frequent clinical/medical follow-up and possibly self-medicate less. This pattern of higher medication use among adults was particularly evident for antipyretics, which are recommended for the symptomatic management of Covid-19 and commonly used by the general population, but also for medications without scientific evidence of efficacy, which may suggest that a weaker connection to routine care favors the use of off-label medications. We also observed greater medication use among participants who reported seeking medical care, although it is not possible to distinguish whether these medications were prescribed or used on their own initiative.

Patterns of medication use according to religion and level of trust in scientists, variables not traditionally analyzed in studies of medication use, highlight the political and social aspects that were central during the Covid-19 pandemic. We found higher frequencies of chloroquine (p = 0.056) and ivermectin (p = 0.058) use among evangelicals; however, these associations were borderline from a statistical standpoint and should be interpreted with caution. The observed differences may reflect sociocultural patterns related to the influence of religious leaders’ discourses^38^. More consistently, individuals who reported low trust in scientists had a higher frequency of off-label medication use, indicating that distrust of scientific institutions may have directly influenced health behavior. This finding is consistent with the literature on the Covid-19 “infodemic”^14^, which highlights the role of misinformation and the politicization of scientific debate, as well as the loss of trust in public institutions and agencies; all of which lead to the adoption of therapeutic practices lacking scientific evidence. Nevertheless, even among participants who reported trusting scientists, the use of these medications was high.

Among the strengths of this study are the population-based design with face-to-face data collection at the national level, and the large sample size, which allowed for analyses using various categories of stratifiers while maintaining precision in the results.

Our analysis also has some limitations. Regarding methodology, the sample included sentinel municipalities, which are generally larger and more developed, implying that caution is warranted when generalizing the findings to less populous municipalities and rural areas. There was also underrepresentation of children and adolescents and of self-identified white individuals, which was offset by weighting procedures. The study sample was restricted to individuals who reported having experienced one or more episodes of Covid-19; therefore, individuals with asymptomatic episodes, mild respiratory symptoms, or who were not tested may have been excluded from the analyses. For obvious reasons, individuals who died from the disease were not included in our retrospective analysis, which may have led us to underestimate the frequencies of medication use, suggesting that the actual situation may be more concerning. There is also evidence of the “prophylactic” use of off-label drugs, which was promoted by various social actors^20,21,28,39^; as our analyses were limited to individuals who reported Covid-19 episodes, such preventive use was not accounted for. Finally, the inability to distinguish between prescribed medications and those used on one’s own initiative prevented us from assessing potential errors in medical recommendations. Given that medication use was more frequent among individuals who sought medical care, it cannot be ruled out that part of this consumption stems from inappropriate prescriptions and medical advice, consistent with extensive evidence of off-label drug recommendations by the Ministry of Health and state and municipal health departments, as well as by organized medical groups. The limitations listed above suggest that the use of such medications was underestimated in the present analysis.

Brazil had a Covid-19 mortality rate well above the global average. Among the various factors explaining this result, the spread of a false sense of security among the population deserves special mention. The widespread promotion of medications that could cure (or even prevent) the disease, without any scientific evidence, helped confuse the population and ultimately encouraged the widespread use of off-label medications. It should also be noted that, as mentioned in the limitations, our estimates may be underestimated. From a public health perspective, our results reinforce the importance of effective communication strategies regarding evidence-based health. The higher frequency of medication use among women, residents of the northern half of the country, evangelicals, and individuals of lower socioeconomic status indicates the need to prioritize these groups in future interventions. These strategies should include clear risk communication, the development of educational campaigns on the rational use of medications, and approaches with broad reach that effectively target these groups, such as engaging community leaders and utilizing widely used digital media.

## Data Availability

The data used in this study are publicly available and can be accessed at: https://epidemio-ufpel.org.br/epicovid/
