# Supplementary material for: Patterns of medication use among individuals with Covid-19 in Brazil: Epicovid 2.0
Source: Rev Saude Publica. 2026 Jul 31;60:e33. doi: 10.11606/s1518-8787.2026060007542 (PMC13405608; doi:10.11606/s1518-8787.2026060007542)
Supplement: Translation

# Translation: Padrões de uso de medicamentos em indivíduos com covid-19 no Brasil: Epicovid 2.0

**Authors:** Romina Buffarini, Cesar Gomes Victora, Pedro Curi Hallal, Mariângela Freitas da Silveira

**Affiliations:** I Universidade Federal de Pelotas. Programa de Pós-graduação em Epidemiologia. Pelotas, RS, Brasil; II Universidade Federal de Pelotas. Centro Internacional de Equidade em Saúde. Pelotas, RS, Brasil; III University of Illinois Urbana-Champaign. Department of Health and Kinesiology. Urbana, IL, United States

**Keywords:** SARS-CoV-2, Coronavírus, Epidemiologia, Uso de Medicamentos, Estudo Transversal

## Abstract

**OBJETIVO:**

Descrever o padrão de uso de medicamentos que os indivíduos com covid-19 usaram para tratar a doença no Brasil, segundo a área de residência, características demográficas, socioeconômicas, religião e nível de confiança em cientistas.

**MÉTODOS:**

Inquérito Nacional Epicovid 2.0 (2024) em 133 municípios. A análise incluiu participantes que relataram ter sofrido episódio de covid-19. Avaliou-se o uso de antibióticos, azitromicina, antitérmicos, cloroquina e ivermectina, além dos desfechos compostos: uso de cloroquina e/ou ivermectina; cloroquina, ivermectina e/ou azitromicina (CIA); e uso de qualquer medicamento. Foram estimadas a frequência de uso e os intervalos de confiança de 95%, considerando o desenho amostral complexo e a ponderação da amostra

**RESULTADOS:**

A frequência de uso de qualquer medicamento para tratar covid-19 foi de 63,0% (IC95% 60,0–65,9). Os antitérmicos foram os mais utilizados (53,7%), seguidos por antibióticos (36,2%) e azitromicina (27,2%). O uso isolado de ivermectina foi relatado por 23,6% e o de cloroquina, por 12,1%; o uso de ivermectina e/ou cloroquina atingiu 27,8% e o CIA, 39,0%. No geral, o consumo de medicamentos foi mais frequente nas regiões Norte, Nordeste e Centro-Oeste e entre mulheres. O uso concentrou-se entre adultos de 40–59 anos, pessoas com baixa escolaridade e menor renda. Pessoas autodeclaradas pretas e pardas relataram maior consumo de cloroquina. Uso de cloroquina, ivermectina e combinações destes medicamentos foi mais comum entre evangélicos (0,05 < p < 0,1) e entre pessoas que consultaram médicos (p < 0,001). Participantes que relataram desconfiar de cientistas relataram maior uso de cloroquina e de cloroquina/ivermectina em comparação com os que relataram confiar em cientistas.

**CONCLUSÃO:**

Quatro em cada dez pessoas que relataram episódios de covid-19, para tratar a doença, consumiram medicamentos não recomendados pela ciência. O uso de medicamentos tendeu a ser mais frequente entre mulheres, na metade norte do Brasil, entre evangélicos e pessoas de menor renda e escolaridade. Esses grupos devem ser priorizados em campanhas futuras sobre o uso adequado de medicamentos.

## INTRODUÇÃO

Os medicamentos constituem insumos centrais das práticas de cuidado em saúde, empregados na prevenção e no tratamento de doenças. Entretanto, seu uso pode estar associado a efeitos adversos relevantes quando realizado de forma inadequada^1^. No início da pandemia de covid-19, diante da ausência de terapias eficazes para um vírus até então desconhecido, diferentes medicamentos com certa plausibilidade biológica para tratar as complicações da doença passaram a ser testados e utilizados na prática clínica por meio de estratégias de reposicionamento terapêutico^2,3^. Nesse contexto, o uso *off-label* foi adotado em diferentes cenários assistenciais. Os avanços das evidências, no entanto, passaram a demonstrar a ausência de benefício para a maioria dessas intervenções^4^.

Apesar disso, ao longo dos meses subsequentes ao início da pandemia, foi observada uma intensa utilização de fármacos tanto sob prescrição médica quanto por iniciativa própria da população^8,9^, frequentemente sem respaldo científico^10^, o que suscitou preocupações quanto ao uso irracional de medicamentos e aos potenciais riscos individuais e coletivos^11^. Além das práticas de uso de medicamentos, como automedicação frequente, já descritas no país antes da pandemia^12,13^, emergiu um cenário marcado pela ampla difusão de informações não baseadas em evidências acerca de supostos tratamentos para a doença^14^.

Análises de padrões de desinformação em redes sociais indicaram o Brasil como um dos países com maior destaque em conteúdos falsos ou enganosos sobre medicamentos recomendados ao longo da pandemia^14^, sugerindo baixa incorporação das evidências científicas no debate público. Enquanto instituições cientificas nacionais e internacionais rechaçavam o uso de vários medicamentos (como cloroquina, ivermectina e azitromicina)^5,7,15^, seu uso foi estimulado pela Presidência da República e pelo Ministério da Saúde^20,21^, e respaldado por entidades médicas, por líderes religiosos evangélicos e por inúmeros influenciadores digitais^22^, configurando a desinformação como estratégia vinculada às disputas políticas no enfrentamento da epidemia^25^. A literatura acadêmica evidencia que a promoção dessas drogas não se deu por acaso, mas constituiu uma estratégia política de gestão da pandemia. Ao oferecer uma “cura”, os grupos promotores – sejam eles estatais, médicos, midiáticos ou religiosos – buscavam evitar medidas de isolamento social e transferência de responsabilidade individual pelo cuidado, criando uma falsa sensação de segurança na população.

Apesar da relevância desse contexto, ainda são escassos os dados nacionais representativos sobre os padrões de consumo desses medicamentos durante a pandemia. As evidências existentes baseiam-se, em sua maioria, em populações específicas (por exemplo: estudantes, enfermeiros, professores)^29^, ou estudos com participantes da população geral com amostras pequenas e delineamentos amostrais heterogêneos^32,33^, limitando a generalização dos achados em nível populacional.

Diante dessa lacuna, o objetivo do presente estudo foi descrever os padrões de uso de diferentes medicamentos (recomendados e não recomendados - *off-label*), que indivíduos que relataram ter sofrido covid-19 no Brasil usaram para tratar a doença, segundo região de residência, características demográficas, socioeconômicas, afiliação religiosa e confiança em cientistas, utilizando dados do inquérito nacional de base populacional Epicovid 2.0.

## MÉTODOS

Este estudo utilizou dados da segunda edição do Inquérito Nacional Epicovid (Epicovid 2.0), conduzido entre março e junho de 2024 em todas as 27 unidades federativas do Brasil. A amostragem foi idêntica à do estudo Epicovid original, realizado em quatro etapas durante 2020 para documentar a progressão da pandemia de covid-19 no país. Detalhes metodológicos são encontrados em outra publicação^34^.

O Instituto Brasileiro de Geografia e Estatística (IBGE) divide o território nacional em 133 regiões intermediárias. Em cada uma delas, a cidade mais populosa foi selecionada como área sentinela. A população elegível consistiu em moradores de domicílios particulares permanentes localizados nos setores censitários urbanos sorteados. Usou-se um processo de amostragem probabilística em múltiplos estágios. Em primeiro lugar, 133 municípios foram selecionados; em segundo lugar, foram selecionados 25 setores urbanos com probabilidade proporcional ao tamanho; a seguir, foram sorteados aleatoriamente dez domicílios dentro de cada setor, utilizando mapas e listagens oficiais do IBGE. Por último, foram registrados todos os moradores presentes no domicílio, sendo um morador selecionado aleatoriamente através de um aplicativo móvel. Em caso de recusa, um segundo morador era sorteado e, se ambos recusassem, a equipe seguia para o domicílio vizinho localizado à direita do domicílio original, enumerando seus moradores e selecionando um para a entrevista. Em domicílios unipessoais com recusa, o próximo domicílio era visitado. O plano amostral resultou em um tamanho de 33.250 indivíduos.

As entrevistas foram presenciais, utilizando um aplicativo móvel aplicado por equipe treinada, contratada por empresa especializada em inquéritos populacionais. A coleta ocorreu de maio a junho de 2024, simultaneamente em todas as cidades, reduzindo vieses temporais e sazonais.

### Variáveis

#### Desfechos

Os desfechos foram o relato de uso de medicamentos para tratar a doença durante o episódio de covid-19, sendo as informações obtidas por meio de perguntas especificas sobre o uso de cada medicamento. A amostra analisada foi restrita a indivíduos que relataram haver sofrido um ou mais episódios de covid-19. Para cada fármaco avaliado, a resposta foi codificada de forma dicotômica (sim/não).

Foram analisados cinco grupos de medicamentos: qualquer antibiótico, azitromicina, antitérmicos (coletados no questionário como “remédios para febre”, expressão utilizada para facilitar a compreensão dos entrevistados), cloroquina e ivermectina. Analisou-se também três desfechos compostos: “uso de cloroquina e/ou ivermectina”, “uso de cloroquina, ivermectina e/ou azitromicina”, denominado CIA, e “uso de qualquer medicamento”. Portanto, as análises foram enfocadas em medicamentos *off-label*, os quais obtiveram ampla visibilidade pública durante a pandemia apesar da ausência de comprovação científica de sua eficácia no tratamento da covid-19, conforme revisões sistemáticas e diretrizes clínicas^2,4,6,35^. Os desfechos compostos de medicamentos *off-label* foram definidos com o objetivo de capturar o uso isolado e combinado desses fármacos, refletindo práticas amplamente difundidas no contexto da pandemia.

O desfecho composto “uso de qualquer medicamento” foi definido como uso de ao menos um dos seguintes: antibióticos, antitérmicos, anti-inflamatórios/corticoides, cloroquina, ivermectina ou nirmatrelvir/ritonavir sendo utilizado para representar o panorama geral do uso de medicamento entre indivíduos com covid-19. A utilização de nirmatrelvir/ritonavir foi inicialmente investigada; contudo, devido à baixa frequência de seu uso (2,2%), esse fármaco não foi incluído nas análises como desfecho individual, permanecendo apenas dentro do desfecho composto “uso de qualquer medicamento”. O antibiótico azitromicina foi investigado separadamente, sendo também incluído nos desfechos CIA, uso de qualquer antibiótico e uso de qualquer medicamento. Sua análise isolada justifica-se por ter sido o antibiótico mais utilizado no contexto da pandemia, além de integrar o grupo de medicamentos amplamente utilizados sem evidência científica de eficácia para tratar a covid-19.

#### Exposições

As variáveis explanatórias incluíram região geográfica de residência, características sociodemográficas, afiliação religiosa, confiança em cientistas e procura por serviço médico. A região de residência foi classificada conforme as macrorregiões brasileiras: Norte, Nordeste, Centro-Oeste, Sudeste e Sul.

As variáveis sociodemográficas incluíram raça-cor autorreferida, segundo as categorias do IBGE (branca, preta, parda, amarela ou indígena); sexo (feminino ou masculino); idade, categorizada em faixas etárias (0 a 19, 20 a 39, 40 a 59, 60 a 69 e 70 ou mais); e escolaridade em anos de estudo completos (0 - sem instrução formal, 1 a 4, 5 a 8, 9 a 12 e 13 anos ou mais). A posição socioeconômica foi estimada com base na renda familiar mensal do domicílio, expressa em salários-mínimos (menos de 1, 1 a 3, 3 a 5 e 5 ou mais).

A afiliação religiosa foi agrupada em três categorias: nenhuma religião, católica e evangélica. Outras religiões (espírita, umbanda/candomblé, judaísmo, islamismo, budismo e outras) não foram analisadas separadamente devido à baixa frequência individual de cada grupo (< 1%) e ao fato de não haver fundamentação conceitual para seu agrupamento, motivo pelo qual foram excluídas das análises específicas dessa variável.

A variável confiança em cientistas foi avaliada pela seguinte pergunta: *“Qual o seu grau de confiança para as informações sobre covid-19 nos cientistas?”* As respostas foram inicialmente coletadas em escala ordinal de cinco pontos: *nunca confio; confio raramente; nem confio nem desconfio; confio moderadamente; confio sempre*, e posteriormente agrupada em três categorias: “Desconfio” (nunca/raramente confio), “Nem confio nem desconfio” e “Confio” (moderadamente/confio sempre). A procura por algum serviço médico durante o episódio de covid (sim/não) também foi incluída.

## Análises

Foram realizadas análises descritivas (frequências absolutas e relativas). As frequências e os intervalos de confiança de 95% (IC95%) dos oito desfechos foram estratificados segundo as variáveis explanatórias.

Para verificar a sobreposição do uso de medicamentos *off-label* (cloroquina, ivermectina e azitromicina), foi elaborado um diagrama de Venn, apresentando as suas combinações de uso isolado e concomitante.

Todas as análises consideraram o desenho amostral complexo (pesos amostrais, amostra estratificada por municípios e uso de conglomerados dentro de cada município) por meio do comando *svy* do *software* Stata. A descrição detalhada do cálculo dos pesos amostrais ponderados é apresentada em publicação específica do protocolo Epicovid 2.0^34^, sendo que todos os resultados apresentados a seguir foram ponderados. Houve dados faltantes apenas para escolaridade (6%) e renda familiar (7%). Os participantes com valores ausentes nessas variáveis não foram incluídos nas análises estratificadas correspondentes. Todas as análises estatísticas foram realizadas no Stata, versão 19.0 (StataCorp, College Station, Estados Unidos).

## Aspectos Éticos

O estudo foi aprovado pelo Comitê de Ética da Escola de Educação Física da Universidade Federal de Pelotas (CAAE: 68382923.8.0000.5313). Todos os participantes adultos assinaram termo de consentimento livre e esclarecido. Para menores de idade, o consentimento foi obtido do responsável legal.

## RESULTADOS

A amostra analítica foi restrita a 9.591 participantes com relato prévio de covid-19, correspondendo a 28,6% (IC95% 27,3–30,0) da amostra total^34,36^. Destes indivíduos, a maioria relatou apenas um episódio (76,7%), sendo que 17% relataram dois, 5% relataram três e 1% disseram ter sofrido quatro ou mais episódios de covid. A frequência de uso de qualquer medicamento foi de 63,0% (IC95% 60,0–65,9). Os medicamentos mais utilizados foram os antitérmicos (53,7%; IC95% 50,8–56,6), seguidos por antibióticos (36,2%; IC95% 33,3–39,2) e azitromicina (27,2%; IC95% 24,7–29,9). O uso isolado de ivermectina foi relatado por 23,6% (IC95% 21,5–25,9) dos participantes e o de cloroquina por 12,1% (IC95% 10,7–13,5). A utilização de ivermectina e/ou cloroquina atingiu 27,8% (IC95% 25,5–30,3), enquanto o desfecho composto CIA (cloroquina, ivermectina e/ou azitromicina) apresentou frequência de 39,0% (IC95% 36,3–41,8) (Figura 1).

**Figura 1:** Prevalências ponderadas de uso de medicamentos entre participantes com relato de covid-19. Epicovid 2.0, Brasil, 2024 (n = 9.591). CIA: cloroquina, ivermectina e/ou azitromicina.

O diagrama de Venn (Figura 2) mostra a sobreposição de uso de cloroquina, ivermectina e azitromicina, evidenciando que dos 40% de participantes que usaram algum dos medicamentos *off-label* e 4,9% usaram os três.

**Figura 2:** Sobreposição do uso de medicamentos *off-label* (cloroquina, ivermectina e azitromicina) entre participantes com covid-19. Epicovid 2.0, Brasil, 2024 (n = 9.591).

A amostra foi composta principalmente por residentes nas regiões Sudeste (43,6%), seguido pelas regiões Nordeste (17,3%) e Sul (16,6%). Mulheres representaram 52,8% da amostra, e a maioria dos participantes tinha idades entre 20 e 39 anos (38,4%) e entre 40 e 59 anos (32,0%). Quase metade se autodeclararam brancos (47,1%), seguido por 41,4% pretos e 10,6% pardos, enquanto as categorias amarela e indígena representaram menos de 1% da amostra. Em relação à escolaridade, quase 80% dos participantes possuíam 9 ou mais anos de educação formal, e 2% relataram não ter nenhuma instrução formal. Dois terços da amostra relataram receber até 3 salários-mínimos, um quinto referiu 3 a 5 salários-mínimos (19,1%) e 13,9%, receberam 5 ou mais. A maioria dos participantes declarou-se católica (39,4%) ou evangélica (35,0%), cerca de 70% relataram confiar em cientistas e ter procurado algum tipo de serviço médico durante o episódio de covid (Tabelas 1 e 2).

No geral, o uso de todos os medicamentos foi mais frequente na região Norte, seguida pelo Nordeste e Centro-Oeste, com frequências menores no Sudeste e Sul (p < 0,001), exceto para o uso de cloroquina, ivermectina e o desfecho combinado de ambas estas drogas, cujas maiores frequências foram evidenciadas no Norte e Centro-Oeste (Tabela 1). As mulheres apresentaram maior uso de antibióticos, antitérmicos, azitromicina, CIA e de qualquer medicamento, em relação aos homens. O uso isolado de cloroquina foi mais frequente entre homens, com diferença limítrofe (p = 0,056). Quanto à idade, as maiores frequências concentraram-se no grupo de 40 a 59 anos, com redução do uso entre os mais jovens e os mais idosos, embora sem padrão uniforme para todos os medicamentos.

**Tabela 1:** Prevalência do uso de medicamentos para tratar covid-19 durante a pandemia segundo região, sexo e idade (resultados ponderados). Epicovid 2.0, Brasil, 2024 (n = 9.591).

Variáveis explanatórias	n^a^ (%)	Antibióticos	Antitérmicos	Cloroquina	Ivermectina	Azitromicina	Cloroquina/ivermectina	CIA	Qualquer remédio
		% (IC95%)	% (IC95%)	% (IC95%)	% (IC95%)	% (IC95%)	% (IC95%)	% (IC95%)	% (IC95%)
Região		p < 0,001	p = 0,008	p < 0,001	p < 0,001	p < 0,001	p < 0,001	p < 0,001	p < 0,001
Norte	1.770 (11,9)	52,3 (46,9–57,7)	63,4 (58,8–67,7)	19,9 (16,8–23,4)	32,7 (28,9–36,7)	41,9 (37,1–46,9)	37,6 (33,5–41,8)	54,0 (49,4–58,6)	74,1 (69,4–78,3)
Nordeste	2.480 (17,3)	38,7 (35,5–42,0)	57,9 (54,0–61,7)	12,1 (10,0–14,6)	29,8 (26,8–33,1)	28,6 (25,7–31,6)	33,5 (30,4–36,7)	43,5 (40,1–47,0)	67,6 (63,9–71,1)
Centro-Oeste	1.042 (10,6)	36,6 (31,8–41,7)	53,1 (45,7–60,4)	17,6 (12,3–24,6)	31,8 (26,3–37,8)	26,0 (21,5–31,0)	37,2 (30,3–44,8)	43,6 (36,5–51,0)	65,2 (58,4–71,4)
Sudeste	2.526 (43,6)	31,5 (25,6–38,2)	51,3 (45,3–57,2)	8,5 (6,5–11,2)	18,3 (14,2–23,3)	24,7 (19,5–30,7)	22,5 (18,1–27,6)	34,2 (28,8–40,1)	59,2 (53,1–65,1)
Sul	1.773 (16,6)	33,9 (30,7–37,3)	49,0 (45,8–52,2)	12,0 (10,2–14,2)	19,4 (17,0–22,2)	22,8 (20,1–25,7)	23,0 (20,5–25,8)	32,9 (30,0–35,9)	58,7 (55,0–62,4)
Sexo		p = 0,003	p = 0,004	p = 0,056	p = 0,1	p < 0,001	p = 0,46	p = 0,002	p = 0,001
Masculino	3.293 (47,2)	31,9 (27,9–36,2)	49,4 (44,7–54,0)	13,5 (11,2–16,3)	21,5 (17,8–25,7)	21,8 (18,2–25,8)	26,8 (22,9–31,2)	34,4 (30,2–38,8)	58,0 (53,4–62,6)
Feminino	6.298 (52,8)	40,0 (36,3–43,9)	57,5 (54,2–60,8)	10,7 (9,4–12,3)	25,5 (23,1–28,0)	32,1 (29,0–35,5)	28,7 (26,1–31,5)	43,1 (39,7–46,5)	67,4 (63,9–70,7)
Faixa etária (anos)		p = 0,47	p = 0,004	p < 0,001	p = 0,003	p = 0,047	p = 0,001	p < 0,001	p = 0,125
0–19	658 (13,1)	35,1 (25,6–46,0)	56,3 (47,4–64,8)	7,1 (4,5–11,1)	20,9 (12,5–33,0)	26,5 (16,9–38,9)	24,6 (15,9–36,2)	37,3 (27,7–47,9)	62,8 (54,3–70,5)
20–39	2.844 (38,4)	34,9 (29,9–40,2)	54,8 (49,3–60,2)	9,5 (7,6–11,9)	18,9 (15,9–22,3)	24,8 (21,0–29,2)	23,0 (19,7–26,7)	34,2 (29,9–38,8)	62,0 (56,5–67,3)
40–59	3.557 (32,0)	39,6 (35,6–43,7)	57,2 (52,8–61,5)	16,7 (14,3–19,5)	31,7 (28,2–35,6)	32,9 (29,3–36,8)	36,3 (32,8–40,0)	47,7 (43,7–51,6)	67,3 (62,8–71,6)
60–69	1.551 (10,1)	33,1 (28,0–38,6)	45,4 (39,6–51,4)	13,6 (10,1–18,0)	21,9 (18,0–26,2)	25,1 (20,6–30,3)	27,0 (22,5–32,0)	37,1 (32,0–42,5)	58,0 (51,6–64,2)
≥ 70	978 (6,5)	34,1 (27,3–41,6)	37,3 (29,4–46,0)	11,6 (7,4–17,7)	19,6 (14,4–26,0)	18,2 (13,6–24,0)	22,1 (16,8–28,5)	30,6 (24,4–37,5)	55,4 (47,0–63,5)

IC95%: intervalo de confiança de 95%; CIA: cloroquina, ivermectina e/ou azitromicina.

^a^ Frequência absoluta apresentada sem ponderação pelos pesos amostrais.

A Tabela 2 mostra que raça-cor esteve associada apenas ao uso de cloroquina (p = 0,001), com maior consumo entre pretos e pardos em comparação aos demais grupos. Em relação à escolaridade, maiores frequências de uso de medicamentos foram concentradas entre indivíduos com 5 a 8 anos de estudo, especificamente para o uso de antibióticos, cloroquina, cloroquina/ivermectina e qualquer medicamento (p < 0,05) (Tabela 2). Quanto à renda familiar, identificou-se uma associação inversa, com redução do uso à medida que a renda aumentava, particularmente para antitérmicos (p = 0,003), cloroquina (p = 0,032) e qualquer medicamento (p = 0,005). Padrão semelhante foi observado para os demais medicamentos, embora sem significância estatística.

**Tabela 2:** Prevalência do uso de medicamentos para tratar covid-19 durante a pandemia segundo características socioeconômicas, religião, confiança em cientistas e procura por serviço médico (resultados ponderados). Epicovid 2.0, Brasil, 2024 (n = 9.591).

Variáveis explanatórias	n^a^ (%)	Antibióticos	Antitérmicos	Cloroquina	Ivermectina	Azitromicina	Cloroquina/ivermectina	CIA	Qualquer remédio
		% (IC95%)	% (IC95%)	% (IC95%)	% (IC95%)	% (IC95%)	% (IC95%)	% (IC95%)	% (IC95%)
Cor/raça		p = 0,476	p = 0,6	p = 0,001	p = 0,674	p = 0,741	p = 0,334	p = 0,655	p = 0,159
Branca	3.340 (47,1)	34,9 (30,3–39,7)	54,7 (50,5–58,8)	9,5 (7,9–11,4)	22,9 (19,0–27,2)	26,2 (22,2–30,6)	26,3 (22,3–30,7)	37,7 (33,5–42,2)	64,8 (60,8–68,6)
Preta	4.412 (41,4)	38,2 (34,5–42,0)	52,9 (48,7–57,1)	15,0 (12,7–17,5)	24,8 (22,3–27,6)	28,1 (25,2–31,1)	30,0 (26,9–33,3)	40,4 (36,9–44,0)	62,0 (57,7–66,2)
Parda	1.244 (10,6)	34,1 (27,9–40,9)	51,5 (44,6–58,3)	12,2 (8,4–17,3)	22,2 (17,9–27,1)	28,5 (22,4–35,4)	26,3 (21,2–32,1)	39,2 (32,5–46,3)	57,9 (51,0–64,4)
Amarela	204 (0,4)	34,9 (22,7–49,5)	50,8 (34,6–66,9)	9,2 (4,8–17,0)	21,7 (12,9–34,2)	23,5 (14,6–35,6)	24,4 (15,0–37,1)	31,0 (20,1–44,4)	68,5 (52,1–81,3)
Indígena	132 (0,5)	40,8 (23,0–61,3)	68,2 (49,0–82,7)	14,7 (6,6–29,8)	25,0 (12,9–43,0)	32,0 (17,6–51,0)	27,4 (14,5–45,6)	36,9 (20,7–56,8)	77,8 (60,2–89,1)
Escolaridade (anos)		p = 0,018	p = 0,081	p = 0,004	p = 0,06	p = 0,392	p = 0,005	p = 0,07	p = 0,008
Sem instrução	301 (2,0)	43,0 (32,0–54,7)	60,6 (49,2–70,9)	14,1 (8,9–21,4)	21,5 (14,7–30,2)	29,2 (20,6–39,7)	27,3 (19,4–36,9)	38,4 (28,5–49,4)	67,8 (56,7–77,2)
1 a 4	919 (6,2)	34,2 (28,3–40,6)	52,4 (44,9–59,7)	12,4 (8,5–17,7)	16,9 (13,1–21,6)	22,2 (17,2–28,0)	22,4 (17,4–28,3)	34,0 (27,8–40,8)	59,6 (51,9–66,8)
5 a 8	1.774 (13,7)	46,3 (38,6–54,2)	61,0 (54,3–67,3)	16,0 (12,1–20,8)	30,0 (21,8–39,7)	32,0 (23,9–41,4)	35,6 (27,4–44,7)	46,8 (38,9–54,8)	72,3 (66,7–77,3)
9 a 12	4.556 (50,9)	35,2 (31,9–38,6)	54,0 (50,3–57,6)	12,9 (10,9–15,2)	24,5 (21,8–27,4)	27,0 (24,1–30,1)	29,6 (26,6–32,8)	39,3 (36,0–42,8)	63,0 (59,1–66,7)
≥ 13	1.984 (27,2)	33,4 (27,5–39,8)	49,9 (43,6–56,2)	8,4 (6,5–10,8)	20,8 (17,0–25,2)	26,7 (21,8–32,3)	22,3 (18,4–26,7)	36,1 (30,8–41,7)	59,4 (53,7–64,9)
Renda familiar (SM)		p = 0,056	p = 0,003	p = 0,032	p = 0,842	p = 0,298	p = 0,174	p = 0,197	p = 0,005
< 1	2.672 (24,5)	42,0 (36,5–47,7)	61,9 (57,1–66,6)	13,7 (11,1–16,8)	24,0 (20,5–27,9)	31,0 (25,9–36,6)	28,7 (24,6–33,3)	43,1 (37,8–48,5)	70,1 (65,5–74,3)
1 a 3	3.891 (42,5)	36,6 (32,0–41,6)	54,4 (49,8–59,0)	13,5 (11,0–16,5)	23,9 (20,0–28,4)	27,4 (23,3–31,9)	30,0 (25,8–34,6)	39,8 (35,5–44,1)	63,8 (59,2–68,2)
3–5	1.510 (19,1)	33,5 (28,2–39,2)	50,2 (43,7–56,6)	10,7 (8,4–13,5)	24,2 (19,6–29,5)	24,5 (20,1–29,5)	26,8 (22,2–32,0)	36,0 (30,8–41,5)	58,6 (52,1–64,9)
≥ 5	927 (13,9)	30,0 (23,0–38,0)	44,8 (36,7–53,2)	8,1 (6,0–11,0)	21,3 (16,9–26,4)	24,1 (17,5–32,2)	22,4 (17,9–27,6)	34,7 (27,6–42,6)	55,1 (46,9–63,1)
Religião		p = 0,123	p = 0,454	p = 0,056	p = 0,058	p = 0,292	p = 0,08	p = 0,08	p = 0,627
Nenhuma	1.646 (25,6)	31,3 (24,3–39,1)	53,9 (46,7–60,9)	10,0 (7,2–13,5)	18,8 (13,1–26,4)	23,3 (16,8–31,4)	24,4 (18,1–31,9)	33,3 (26,3–41,1)	62,3 (55,4–68,7)
Católica	4.316 39,4)	37,6 (33,8–41,5)	51,6 (47,8–55,4)	12,2 (10,3–14,5)	23,6 (20,8–26,7)	28,1 (24,7–31,8)	27,0 (24,0–30,2)	40,1 (36,4–43,9)	62,4 (58,5–66,2)
Evangélica	3.058 (35,0)	38,8 (34,9–42,9)	55,8 (51,2–60,3)	15,1 (12,3–18,3)	27,7 (24,6–31,0)	29,0 (25,5–32,7)	32,6 (28,8–36,6)	42,3 (38,0–46,8)	65,2 (60,7–69,4)
Confiança em cientistas		p = 0,136	p = 0,41	p < 0,001	p = 0,349	p = 0,751	p = 0,022	p = 0,232	p = 0,199
Confio	6.283 (70,7)	34,6 (30,8–38,6)	53,9 (50,1–57,7)	10,1 (8,7–11,8)	22,8 (19,9–25,9)	26,8 (23,3–30,6)	26,2 (23,2–29,4)	37,9 (34,2–41,6)	62,3 (58,5–66,0)
Nem confio, nem desconfio	1.434 (13,9)	39,9 (33,2–47,1)	50,0 (43,0–57,0)	12,4 (9,4–16,3)	25,5 (20,9–30,7)	28,4 (23,3–34,1)	29,3 (24,3–34,8)	39,6 (33,6–45,9)	60,8 (53,5–67,6)
Desconfio	1.874 (15,4)	40,1 (35,7–44,7)	56,0 (51,1–60,7)	20,6 (16,8–25,0)	25,7 (22,6–29,2)	28,4 (24,9–32,2)	34,0 (29,5–38,9)	43,5 (39,0–48,2)	68,0 (63,2–72,5)
Procura de serviço médico		p < 0,001	p < 0,001	p < 0,001	p < 0,001	p < 0,001	p < 0,001	p < 0,001	p < 0,001
Não	3.021 (30,9)	21,2 (18,2–24,7)	36,6 (32,4–41,1)	8,3 (6,5–10,6)	16,4 (14,0–19,2)	16,4 (13,6–19,7)	18,8 (16,1–21,8)	26,1 (22,6–29,9)	46,1 (41,6–50,7)
Sim	6.750 (69,1)	42,9 (39,2–46,6)	61,3 (58,1–64,4)	13,7 (12,0–15,6)	26,8 (24,0–29,9)	32,1 (28,9–35,4)	31,9 (28,9–35,0)	44,7 (41,6–47,9)	70,5 (67,4–73,5)

IC95%: intervalo de confiança de 95%; CIA: cloroquina, ivermectina e/ou azitromicina; SM: salário-mínimo.

^a^ Frequência absoluta apresentada sem ponderação pelos pesos amostrais.

Quanto à religião, verificaram-se frequências mais elevadas entre indivíduos evangélicos para a maioria dos medicamentos analisados, embora associações próximas à significância estatística tenham sido observadas apenas para cloroquina (p = 0,056), ivermectina (p = 0,058) e para os desfechos combinados (p = 0,08).

Os indivíduos que relataram desconfiar de cientistas apresentaram frequência de uso de cloroquina aproximadamente duas vezes maior (p < 0,001) e de cloroquina/ivermectina 1,3 vezes maior (p = 0,022), em comparação aos que relataram confiar em cientistas. O uso de todos os medicamentos foi maior entre indivíduos que procuraram algum serviço médico durante o episódio de covid (p < 0,001).

## DISCUSSÃO

Por sua abrangência nacional, o presente estudo fornece o mais completo painel do uso de medicamentos durante episódios de covid-19 no Brasil, e possivelmente o mais amplo estudo em nível mundial. Nosso objetivo foi medir padrões de utilização conforme diversas variáveis estratificadoras, por meio de análises simples e descritivas, com o objetivo de subsidiar políticas de saúde para futuras epidemias. Por este motivo, não houve indicação para análises mais complexas ou modelos estáticos multivariáveis.

Nossos resultados evidenciaram diferenças regionais, sociodemográficas e comportamentais no uso de medicamentos recomendados – assim como de medicamentos *off-label* - entre os participantes que relataram covid-19. Quase dois terços usaram pelo menos um tipo de medicamento, e cerca de 40% utilizaram um ou mais *off-label* como cloroquina, ivermectina e/ou azitromicina.

Em linhas gerais, os padrões de uso de medicamento por região, sexo e faixa etária identificados neste estudo são consistentes com a literatura brasileira sobre automedicação em condições agudas^12,37^. O estudo de base populacional: Pesquisa Nacional sobre Acesso, Utilização e Promoção do Uso Racional de Medicamentos (PNAUM) realizada entre setembro de 2013 e outubro de 2014 evidenciou em diferentes análises, maior consumo de medicamentos nas mulheres, além de importantes desigualdades regionais e socioeconômicas no uso desses insumos. O maior uso observado nas regiões Nordeste, Centro-Oeste e Norte reproduz os achados da PNAUM e pode refletir desigualdades regionais no acesso aos serviços de saúde^12,37^, com maior automedicação em contextos de menor oferta de cuidados formais. De forma semelhante, o maior consumo de medicamentos entre indivíduos de menor renda evidenciado no nosso estudo, pode estar influenciado pelo menor acesso a cuidado médico oportuno em grupos socioeconomicamente mais vulneráveis.

As mulheres apresentaram maior uso da maioria dos medicamentos analisados, resultado já descrito nas análises prévias da PNAUM^12,37^; a única exceção foi o maior uso de cloroquina entre homens, o qual pode estar relacionado a aspectos da promoção desta droga, que serão discutidos abaixo. Quanto à idade, o consumo de todas as categorias de medicamentos analisadas foi maior entre adultos de 40 a 49 anos, este achado mostra concordância com a análise da PNAUM que avaliou a automedicação no Brasil e mostrou que a prevalência de uso de medicamentos por contra própria foi maior nas faixas etárias de 20 a 39 e 40 a 59 anos em comparação a grupos de idades extremos^12^. Este fato pode refletir diferenças na utilização dos serviços de saúde, uma vez que indivíduos mais velhos geralmente têm acompanhamento clínico/médico mais frequente e possivelmente se automedicam menos. Esse padrão de uso de maior uso de medicamento entre adultos foi particularmente evidente para os antitérmicos, recomendados para o manejo sintomático da covid-19 e normalmente usados pela população, mas também para medicamentos sem evidência científica de eficácia, o que pode sugerir que um menor vínculo com o cuidado de rotina favoreceria o uso de medicamentos *off-label*. Também observamos maior uso de medicamentos entre os participantes que relataram ter procurado serviço médico, embora não seja possível distinguir se esses medicamentos foram prescritos ou utilizados por iniciativa própria.

O padrão de uso de medicamentos conforme religião e nível de confiança em cientistas, variáveis tradicionalmente não analisadas em estudos de uso de medicamentos, evidenciam os aspectos políticos e sociais que foram centrais durante a pandemia de covid-19. Encontramos maiores frequências de uso de cloroquina (p = 0,056) e ivermectina (p = 0,058) entre evangélicos; no entanto, essas associações foram limítrofes do ponto de vista estatístico, devendo ser interpretadas com cautela. As diferenças observadas podem refletir padrões socioculturais relacionados à influência de discursos de líderes religiosos^38^. De forma mais consistente, indivíduos que relataram baixa confiança em cientistas apresentaram maior frequência de uso de medicamentos *off-label*, indicando que a desconfiança em instituições científicas pode ter influenciado diretamente o comportamento em saúde. Esse achado está alinhado com a literatura sobre a “infodemia” da covid-19^14^, que destaca o papel da desinformação e politização do debate científico, e a perda de confiança em instituições e agência públicas; que levam à adoção de práticas terapêuticas sem evidência científica. Ainda assim, mesmo entre os participantes que relataram confiar em cientistas, o uso desses medicamentos mostrou-se elevado.

Entre os pontos positivos do presente estudo, destacam-se o delineamento de base populacional com coleta de dados face-a-face em nível nacional, e o grande tamanho amostral que possibilitou análises utilizando diversas categorias de estratificadores e mantendo precisão nos resultados.

Nossas análises também apresentam algumas limitações. Em relação à metodologia, a amostra incluiu municípios sentinela, geralmente maiores e mais desenvolvidos, o que implica cautela na generalização dos achados para municípios menos populosos e para áreas rurais. Também houve sub-representação de crianças e adolescentes e de indivíduos autodeclarados brancos, que foi compensada pelos procedimentos de ponderação. A amostra do estudo foi restrita a indivíduos que relataram ter sofrido um ou mais episódios de covid-19; portanto, indivíduos com episódios assintomáticos, sintomas respiratórios leves ou que não foram testados podem ter sido deixados fora das análises. Por motivos óbvios, indivíduos que faleceram devido à doença não foram incluídos em nossa análise retrospectiva, o que pode ter nos levado a subestimar as frequências de uso de medicamentos, sugerindo que o cenário real possa ser mais preocupante. Há também evidências do uso “profilático” de drogas *off-label*, o qual foi promovido por diversos atores sociais^20,21,28,39^; como nossas análises foram limitadas a indivíduos que relataram episódios de covid-19, tal uso preventivo não foi contabilizado. Finalmente, a impossibilidade de distinguir medicamentos prescritos daqueles utilizados por iniciativa própria não nos permitiu avaliar potenciais erros nas recomendações médicas. Considerando que o uso de medicamentos foi mais frequente entre aqueles indivíduos que procuraram por algum serviço médico, não pode se descartar que parte desse consumo venha de prescrições e aconselhamento médico inadequado, em consonância com amplas evidências da recomendação de drogas *off-label* pelo Ministério da Saúde e secretarias de saúde estaduais e municipais, assim como por grupos médicos organizados. As limitações listadas acima sugerem que o uso de tais medicamentos foi subestimado nas presentes análises.

O Brasil apresentou uma mortalidade por covid-19 muito acima da média mundial. Entre os diversos fatores que explicam esse resultado, a propagação de uma sensação de segurança na população deve ser destacada. A ampla promoção de medicamentos que poderiam curar (ou até prevenir) a doença, sem qualquer evidência científica, ajudou a confundir a população e acabou estimulando o amplo uso de medicamentos *off-labels*. Destaca-se ainda que, conforme mencionado nas limitações, nossas estimativas podem estar subestimadas. Do ponto de vista da saúde pública, nossos resultados reforçam a importância de estratégias de comunicação efetivas no que se refere a saúde baseada em evidência. A maior frequência de uso de medicamentos entre mulheres, moradores da metade norte do Brasil, evangélicos e indivíduos de menor nível socioeconômico, indicam a necessidade de priorização desses grupos em futuras intervenções. Essas estratégias devem incluir ações de comunicação de risco claras, desenvolvimento de campanhas educativas sobre o uso racional de medicamentos e abordagens com ampla capilaridade, que alcancem efetivamente esses grupos, como o envolvimento de lideranças comunitárias e o uso de mídias digitais amplamente utilizadas.

## Data Availability

Os dados utilizados neste estudo são de acesso público e encontram-se disponíveis em: https://epidemio-ufpel.org.br/epicovid/
